# Gla-Rich Protein Is Associated with Vascular Calcification, Inflammation, and Mineral Markers in Peritoneal Dialysis Patients

**DOI:** 10.3390/jcm13237429

**Published:** 2024-12-06

**Authors:** Catarina Marreiros, Carla Viegas, Anabela Malho Guedes, Ana Paula Silva, Ana Catarina Águas, Marília Faísca, Leon Schurgers, Dina Costa Simes

**Affiliations:** 1 Centre of Marine Sciences, University of Algarve, 8005-139 Faro, Portugal; cimarreiros@ualg.pt (C.M.); caviegas@ualg.pt (C.V.); 2 GenoGla Diagnostics, CCMAR, Universidade do Algarve, 8005-139 Faro, Portugal; 3 Unidade local de Saúde do Algarve, Centro Hospitalar Universitário do Algarve (CHUA), 8000-386 Faro, Portugal; anabelamalho@hotmail.com (A.M.G.); anapassionara@gmail.com (A.P.S.); 4 Faculdade de Medicina e Ciências Biomédicas, Universidade do Algarve, 8005-139 Faro, Portugal; 5 Serviço Radiologia, Centro Hospitalar Universitário do Algarve (CHUA), 8000-386 Faro, Portugal; acaguas@chua.min-saude.pt; 6 SYNLAB Algarve, 8000-307 Faro, Portugal; marilia.faisca@synlab.pt; 7 Department of Biochemistry and Cardiovascular, Maastricht University, 6229 HX Maastricht, The Netherlands; l.schurgers@maastrichtuniversity.nl

**Keywords:** peritoneal dialysis, vascular calcification, Adragão score, Gla-rich protein, biomarker

## Abstract

**Background/Objectives**: Vascular calcification (VC) is a crucial risk factor for cardiovascular diseases (CVD), particularly in chronic kidney disease (CKD) populations. However, the specific relationship between VC and end-stage renal disease (ESRD) patients undergoing peritoneal dialysis (PD) remains to be fully understood. The identification of new biomarkers to improve VC diagnosis and monitoring would significantly impact cardiovascular risk management in these high-risk patients. Gla-rich protein (GRP) is a VC inhibitor and an anti-inflammatory agent and thus is a potential VC marker in CKD. Here we explored the potential role of GRP as a marker for CVD and investigated the impact of VC in 101 PD patients. **Methods**: Circulating total Gla-rich protein (tGRP) was quantified in serum and in 24 h dialysate samples. VC score (VCS) was determined using the Adragão method. **Results:** Serum tGRP was negatively associated with VCS, serum calcium (Ca), phosphate (P), and high-sensitivity C-reactive protein (hsCRP), while it was positively associated with magnesium (Mg). A total of 35.6% of PD patients presented with extensive calcifications (VCS ≥ 3), and the lowest tGRP serum levels were present in this group (419.4 ± 198.5 pg/mL). tGRP in the 24 h dialysate was also negatively associated with VCS and with serum Ca and P. Moreover, serum Ca, P, and VCS were identified as independent determinants of serum tGRP levels. **Conclusions**: The association of serum tGRP with VC, mineral, and inflammation markers reinforces its potential use as a novel VC biomarker in CKD patients undergoing PD.

## 1. Introduction

Cardiovascular disease (CVD) represents a significant burden in chronic kidney disease (CKD) patients, being the leading cause of death in end-stage renal disease (ESRD), for which mortality rates are considerably higher compared to the general population [1]. The relationship between CKD and CVD results in a complex interplay that poses considerable challenges in patient clinical management. Although the mechanisms resulting in increased cardiovascular risk are multifactorial, the poor CVD outcomes are, in part, due to vascular calcification (VC) [2]. VC refers to the abnormal deposition of calcium and phosphate in the blood vessels, particularly in the form of hydroxyapatite crystals [3]. This slow process leads to the hardening and stiffening of vessels, culminating in serious CVD, such as peripheral arterial disease and ventricular hypertrophy, and ultimately leading to fatal events, such as heart attack and stroke [4].

Although the relationship between VC and patients on hemodialysis (HD) has been widely established, the clinical burden of VC in PD patients remains to be explored. Comparisons of the progression of coronary artery calcification between HD and PD patients did not find significant differences in the two types of dialysis modalities, suggesting similar effects on VC development [5]. However, a meta-analysis comparing CVD events between HD and PD patients found that while PD patients have fewer cardiovascular events, they exhibit higher CVD mortality rates than HD patients [6]. Nevertheless, the limited number of studies specifically assessing VC in PD patients is insufficient to currently retrieve any conclusions regarding the effect of dialysis modality on VC burden.

Although VC of the intima and media vessel layer can coexist, calcification of the media is generally the type of calcification commonly observed in CKD patients [7]. The Adragão score is a radiograph method previously demonstrated to have good performance in detecting such types of calcifications [8], combining several clinical advantages, such as a quick, non-invasive, and low-cost assessment. Furthermore, KDIGO guidelines [9] posit that VC assessments, such as radiograph methods, must be part of the routine follow-up of CKD from Stage 3. In concordance with these guidelines, observational studies have demonstrated the prognostic importance of monitoring and diagnosing VC in ESRD patients using X-rays [10,11]. However, VC clinical diagnosis and monitoring can be difficult due to the fact that VC is able to remain clinically silent for years, generally disguised by aging [1]. In addition, some CKD cohorts report that up to 40% of patients seem to display clinically undetectable VC [12,13,14,15], despite the existence of well-known triggers. Such data seem to suggest that imaging biomarkers may display limitations if used alone and that VC diagnosis and management need to be complemented with more clinical information, including circulating VC biomarkers. One mechanism to regulate pathological vascular calcification (VC) is its active inhibition. Naturally occurring VC inhibitors, such as vitamin K-dependent proteins (VKDP), present in circulation, may represent a valuable role in complementing VC diagnosis [16,17,18,19,20,21]. Among these, Gla-rich protein (GRP), which is also known as unique cartilage matrix-associated protein (UCMA), is a VKDP previously that has been shown to play an important role in ectopic calcification [22]. GRP is a small-sized protein (9.5 kDa) comprising 15 potential gamma-carboxylation sites in its 74-amino acid mature protein sequence [23]. It acts as a calcification inhibitor through the inhibition of calcium-phosphate mineral maturation and growth in circulation and the vasculature [22] and by antagonizing bone morphogenetic protein-2, which regulates osteoblast differentiation [24]. In addition to its calcification-inhibitory properties, GRP has also been reported to act as an anti-inflammatory agent [25]. Recently, observational studies have demonstrated that decreased total circulating GRP forms (tGRP) associated with VC in Stage 2–4 CKD [13,26] and CVD cohorts [27]. However, associations between VC and tGRP with ESRD have never been investigated before. Furthermore, there is a critical need for more focused research to understand the specific factors contributing to VC, aiming to develop effective strategies for the diagnosis, prevention, and management of CKD [1].

Hence, the main purpose of this study is to explore VC in PD patients and examine the relationship among tGRP, VC, and serum factors associated with CVD in these patients. This research aims to evaluate the potential of circulating tGRP as a rapid and non-invasive biomarker for integrating VC diagnosis in end-stage CKD.

## 2. Materials and Methods

### 2.1. Study Design

**Figure 1:**
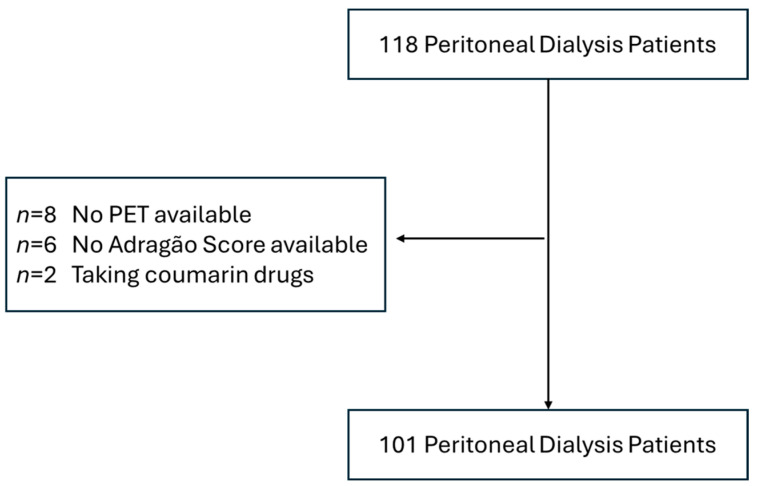
Strobe diagram. Total number of peritoneal dialysis patients included in this study. Abbreviations: PET, peritoneal equilibrium test.

This cross-sectional study included 101 out of 118 Stage 5 CKD patients undergoing PD (Figure 1). Patients were recruited from the outpatient nephrology department of Unidade Local de Saúde do Algarve (ULSA), Portugal, from March 2015 until March 2022. Inclusion criteria comprised a modified peritoneal equilibration test (PET) using 3.86/4.25% glucose solutions. All patients were treated with neutral pH low-glucose degradation product solutions (Baxter^®^ or Fresenius^®^). Patients without vascular calcification assessments (Adragão score) or under coumarin-derived drug therapy were excluded. Informed consent was obtained from all the participants. Demographic and clinical data were retrieved from the hospital medical records. This study followed the STROBE checklist, was in compliance with the Declaration of Helsinki, and was approved by local ethics committee.

### 2.2. Samples Handling and Biochemical Measurements

Blood samples were obtained from the antecubital vein in resting conditions, with the subject’s arm in a supine position on the same day of PET. Serum samples were prepared using standard centrifugation, following serum handling considerations before storage at −80 °C until further use. Serum determinations were assessed using automated analysis on an ADVIA^®^ 1800 Clinical Chemistry System SIEMENS^®^, (Synlab, Faro, Portugal). A 24 h dialysate and urine were collected during PET on the same day of the blood sample harvesting. Residual kidney function (RKF) refers specifically to the remaining endogenous renal function and was obtained through the average clearance of urea and creatinine from urine. Creatinine clearance reflects the combined clearance achieved by both RKF and the dialysis process and was calculated on a weekly basis to ensure dialysis adequacy [28]. Total GRP (tGRP) serum levels were determined using a previously validated sandwich ELISA kit (GenoGla Diagnostics, Faro, Portugal) [22]. For all samples, 3 independent tGRP quantifications were performed on different days by the same operator. The quantification of tGRP for each sample resulted from the mean of the triplicates obtained from these three independent runs. Inter- and intra-assay variability for tGRP quantifications were found to be approximately 3% and 5%, respectively. Serum calcium (Ca) was corrected for albumin serum levels using Jain et al.’s formula, as follows: corrected Ca (mg/dl) = Ca (mg/dl) + 0.8 × [3 − serum albumin (g/dL)] [29]. Calcium(x)phosphate product (CaxP) was calculated through the direct product of each individual (Ca) and (P) measurements.

### 2.3. Medical Assessments

Body mass index (BMI) was calculated through the equation weight/height^2^ (kg/m^2^). VC was performed using plain X-rays on the hands and pelvis, as described [8]. The vascular calcification score (VCS) was determined using the Adragão method and validated by an experienced and trained radiology specialist, who was blinded to other results. The patients were divided into the following three groups according to VCS: (a) VCS = 0, which was considered undetectable or absent calcification; (b) 1 ≤ VCS ≤ 2, ranging between 1 and 2; and (c) VCS ≥ 3, which was considered increased cardiovascular risk [8,30,31,32].

### 2.4. Statistical Analysis

The data normality was verified using visual (histograms, Q-Q plots) and analytic methods (Kolmogorov–Smirnov/Shapiro–Wilk tests). All variables had a normal distribution and are presented as mean and standard deviation. Categorical variables are presented as absolute frequencies (*n*) and relative frequencies (percentages, %). For non-normally distributed continuous variables (hsCRP), a logarithmic transformation was applied to approximate normality. The differences between the two groups were evaluated with the appropriate significance test (e.g., Student’s *t*-test). For comparisons between VCS groups in PD populations, one-way ANOVA tests with Tukey’s post hoc analyses were applied to determine differences in serum measurements. Pairwise correlations to check for possible associations between quantitative variables were performed using Pearson’s correlation coefficient. Linearity and normality were verified, and variables showing significant correlations with serum tGRP in Pearson correlation analysis (*p* < 0.05) were included in a stepwise linear regression analysis to identify the most significant predictors of tGRP.

A statistical analysis was conducted using the Statistical Package for the Social Sciences (SPSS v.26, Inc., Chicago, IL, USA), and plots were built using GraphPad Prism version 8 (GraphPad Software, San Diego, CA, USA) for Windows software. Significance was set at *p* ≤ 0.05.

## 3. Results

### 3.1. Population Characterization

Clinical and demographic data for peritoneal dialysis patients (*n* = 101) are presented in Table 1. Overall, the mean age was 53.11 ± 16.18 years, ranging from 18 to 90 years old. A higher representation of the male gender (*n* = 62; 61.4%) was observed, and an average overweight BMI was found (25.03 ± 0.53) for the total population. Clinically relevant biochemical parameters revealed a heightened lipid profile, which was given by total cholesterol and LDL-C values of 210 ± 26.21 mg/dL and 135 ± 30.1 mg/dL respectively. An increased inflammatory state was observed, which was reflected by an average hsCRP value of 8.82 ± 12.36 mg/L [33]. Mean quantifications of serum mineral markers were 5.11 ± 1.65 mg/dL for P; 7.88 ± 1.07 mg/dL for Ca; 36.67 ± 3.08 mg/dL for CaxP; and 2.13 ± 0.39 mg/dL for Mg. Mean serum levels for tGRP were 442.3 ± 34.02 pg/mL, ranging from 37 to 1450 pg/mL (minimum to maximum).

Specific data regarding dialysis and peritoneal equilibrium test (PET) were also retrieved (Table 1). The average time on dialysis for these patients was 27.7 ± 2.97 months. Dialysis characterization showed high icodextrin use (53.5%), with only 32.7% of PD patients on continuous ambulatory peritoneal dialysis (CADP). Moreover, tGRP was also detected and quantified in the 24 h dialysate fluid at a concentration of 108 ± 10.21 pg/mL, ranging from 0.33 to 480.3 pg/mL (minimum to maximum). tGRP concentrations in 24 h dialysate were approximately four times lower than in serum (442 pg/mL to 108 pg/mL). Vascular calcification was present in 59.4% of patients, with an overall VCS mean of 1.91 ± 2.18. A total of 35.6% of the population (*n* = 36) presented with extensive calcifications, with VCS values ≥ 3 (*n* = 24 males and *n* = 12 females); 23.8% had VCS values ranging between 1 and 2, and 41.2% had scores indicating undetectable VC or the absence of VC (VCS = 0).

### 3.2. Serum tGRP Related to Serum Mineral Markers, Vascular Calcification, and Systemic Inflammatory State

To explore the possible associations between tGRP levels in serum and both demographic and biochemical parameters, Pearson’s analysis was performed (Table 1). tGRP serum levels did not correlate with age (r = −0.153; *p* = 0.116), BMI (r = −0.065; *p* = 0.514), lipid profile given by total cholesterol (r = 0.016; *p* = 0.831), HDL-C (r = 0.024; *p* = 0.712), LDL-C (r = 0.091; *p* = 0.614), serum creatinine (r = −0.151; *p* = 0.081), total protein (r = 0.204; *p* = 0.061), or albumin (r = −0.052; *p* = 0.605). However, the results revealed significant negative correlations between tGRP and P (r = −0.482; *p* = 0.001), Ca (r = −0.503; *p* < 0.001), and CaxP (r = −0.575; *p* < 0.001) and a positive association with Mg (r = 0.242; *p* = 0.015). Regarding the inflammatory marker, hsCRP, a negative correlation was observed (r = −0.313; *p* < 0.001). Overall, increased serum mineral markers and the highest inflammatory states were associated with lower tGRP serum levels in these patients. Moreover, the quantification of tGRP in serum and 24 h dialysate correlated positively (r = 0.397; *p* < 0.001), suggesting that patients with higher tGRP serum levels were likely to display increased tGRP in the peritoneal fluid. Pearson’s correlations between serum tGRP and VCS also showed a negative correlation between these parameters (r = −0.409; *p* < 0.001), revealing that patients with increased calcifications presented with lower serum tGRP (Table 1). Student’s *t*-test revealed no differences in serum tGRP between sexes (*p* = 0.501), with icodextrin use (*p* = 0.068), or based on the type of dialysis (*p* = 0.461).

### 3.3. Vascular Calcification, Calcium, and Phosphate Are Independent Predictors of tGRP Serum Variability

In a stepwise linear regression analysis, all the determinants with significant associations with serum tGRP were evaluated as potential factors influencing this variable in the PD population. Only P (*p* = 0.016), Ca (*p* < 0.001), and VCS (*p* < 0.011) retained a significant association with serum tGRP (Table 2). According to the model, these three variables explained 32.7% of the variability in tGRP serum levels (Table 2), with Ca being the strongest independent factor, with an impact of 23.3% in tGRP variability.

### 3.4. tGRP in 24 h Dialysate Correlates with Mineral Serum Markers

Given the associations found for serum tGRP with mineral serum markers and inflammation, we further evaluated whether the same would be observed for 24 h dialysate tGRP levels. Pearson’s analysis revealed that tGRP in 24 h dialysate also correlated negatively with Ca (r = −0.252; *p* < 0.01), P (−0.235; *p* < 0.05), and CaxP (r = −0.324; *p* < 0.01). However, the associations found between serum tGRP and hsCRP (r = 0.017; *p* = 0.831) and Mg (r = 0.172; *p* = 0.071) were lost for tGRP in 24 h dialysate (Table 3).

### 3.5. Vascular Calcification Score Correlates with tGRP in Serum and 24 h Dialysate and with Serum Mineral Markers

Considering the importance of vascular calcifications in the context of ESRD and the understudied relationship between VC and PD patients, additional associations between VC and clinical parameters in this population were explored (Table 4). In addition to the negative correlation between serum tGRP and VCS, the results revealed positive associations between VCS and the serum mineral markers P (r = 0.241; *p* = 0.002), Ca (r = 0.210; *p* < 0.023), and CaxP (r = 0.301; *p* = 0.001) and a negative correlation between VCS and 24 h dialysate tGRP (r = −0.212; *p* = 0.036).

Associations observed between VCS and P, Ca, CaxP, tGRP serum, and tGRP 24 h dialysate were further explored across the three VCS subgroups, as shown in Table 5. Serum mineral markers (P, Ca, and CaxP) and serum tGRP differed between VCS groups (*p* = 0.014; *p* = 0.032; *p* = 0.012; and *p* < 0.001, respectively). tGRP in 24 h dialysate decreased with VCS progression, but the differences between VCS groups were not significant (*p* = 0.211). Patients with extensive vascular calcifications (VCS ≥ 3) presented decreased serum tGRP (428.6 ± 247.5 pg/mL) compared to patients with VCS = 0 (774.3 ± 381.0 pg/mL). Overall, the increase in VCS was followed by the upraise of mineral markers and the reduction of tGRP serum levels (Table 5).

Post hoc comparisons among the VCS groups revealed that the variables P, Ca, CaxP, and serum tGRP significantly differed between the non-detectable/absent calcification (VCS = 0) and the extensive calcification (VCS ≥ 3) groups. The only variable that showed an extra-significant difference between groups was serum tGRP, which differed between the 1 ≤ VCS ≤ 2 and VCS ≥ 3 groups (Table 6).

## 4. Discussion

To the best of our knowledge, this is the first study assessing tGRP levels in renal patients undergoing PD and reporting the presence of tGRP in human peritoneal fluid. In this cross-sectional study, VC was shown to be increased in patients with lower levels of serum tGRP and higher serum levels of Ca, P, and CaxP. Further, serum tGRP correlated with multiple CVD risk markers, (P, Ca, CaxP, Mg, VCS, and hsCRP) and was dependent on Ca, VCS, and P.

Medical evidence demonstrates that VC is a clinical feature of CKD populations, progressing throughout CKD stages, and is considered to be significantly present in ESRD [2]. However, few studies have addressed VC in PD populations. This may be due to the relatively recent medical guidelines and recommendations to choose PD as the first treatment option [34]. Although some studies suggested that PD patients may have worse CVD prognoses compared to HD patients [6], others indicate that differences in VC outcomes may be less pronounced [35,36]. One particular study assessing VC using X-rays in 152 dialysis patients (100 HD and 52 PD) demonstrated that VC was more prevalent in HD than in PD at baseline and even after 1 year of follow-up [10]. Whether dialysis modality may represent a legitimate factor for VC development is currently under debate. One feature that can contribute to uncertainty in this issue is the considerably low number of studies addressing VC in PD compared to HD patients. In the present study, we observed that 35.3% of PD patients had increased CVD risk (VCS ≥ 3). Considering the same VC detection methodology, our observation is that the risk is lower than other reports have suggested for HD and PD patients. A study enrolling 123 HD patients demonstrated an incidence of 52.8% VCS ≥ 3 [8], while another study with 100 HD patients showed a 40.0% incidence of VCS ≥ 3 [10]. For PD patients, a study reported 76.2% of patients with VCS ≥ 3 [32]. Using the same VC detection method, these results reflect different VC incidences in either PD or HD patients, possibly due to the multifactorial nature of VC across CKD populations with different specificities. In addition, we also report a remarkable percentage of 40.6% of PD patients with undetected VC or the absence of VC (VCS = 0), despite the presence of known triggers associated with end-stage renal disease [37]. This is consistent with other reports describing similar results using the Adragão score [13,38] and Agatston score [39]. The difficulty that VC diagnosis and monitoring poses to the clinical routine may reflect these dispersed VC incidences. Since imaging-based methods to assess VC can display limitations in flawlessly detecting calcium deposits [40,41], particularly in early VC, the use of biochemical parameters related to mineral metabolism may be an added value to help examine VC.

In CKD, disturbed mineral metabolism, particularly increased serum P levels, is a significant risk factor for VC, making hyperphosphatemia and elevated calcium-phosphate ionic product early markers of calcification and predictors of CVD risk in CKD patients [2]. In this study, the analysis of PD patients stratified by VCS subgroups revealed that serum tGRP levels decreased as mineral markers (P, Ca, and CaxP) increased, closely tracking the progression of calcification. This observation suggests that GRP has a biological relationship with these clinical parameters. Furthermore, patients with the highest extension of calcification presented the lowest levels of serum tGRP (419.4 ± 198.5 pg/mL) of the overall population, supporting the known role of GRP in VC regulatory mechanisms [22]. Among the parameters analyzed (Ca, P, CaxP, and tGRP), only serum tGRP could significantly differentiate early (1 ≤ VCS ≤ 2) from extensive calcification (VCS ≥ 3), highlighting its higher sensitivity in detecting calcification progression compared to traditional mineral markers. Hypomagnesemia has also been suggested as a risk factor and accelerator of VC in CKD [1]. Although its true impact on CVD risk is still debatable, patients who exhibited lower tGRP had associated decreased circulating Mg serum levels.

Previous data on both CKD and CVD patients demonstrated a relationship between GRP and vascular damage, which was assessed using valvular and vascular calcifications [13,26,27]. Here, we extended this research and further showed that serum Ca was the variable that most accurately determined serum tGRP variability. These results are in full agreement with previous research demonstrating a close relationship between the mineralization processes and GRP [22] through its function as an inhibitor of mineral maturation and growth and a modulator of VSMC osteogenic differentiation [24].

However, other studies have shown no significant relationship between GRP and Ca or P in 35 hemodialysis patients [42]. Controversially, another study demonstrated that GRP was positively associated with carotid intima thickness in a cohort of 106 Stage 3–5 non-dialysis CKD patients [43]. These studies differed in the clinical contexts, addressing different age groups, and, importantly, since different methodological approaches were used for quantifying GRP serum levels, result interpretations and study analyses should be conducted with caution.

Recently, our group reported that in a cohort of 80 diabetic Stage 2–4 CKD patients, serum tGRP declined with renal deterioration throughout the CKD stages [13]. The levels of tGRP for Stage 4 CKD patients were approximately 500 pg/mL, and they were 900 pg/mL for the overall CKD population. In concordance, the present study demonstrated even lower tGRP serum levels in ESRD patients undergoing PD (442.3.3 ± 34.02 pg/mL), suggesting that serum tGRP may be decreased in later CKD stages.

We also demonstrated that decreased serum tGRP is associated with an increased systemic inflammatory profile given by hsCRP. GRP was shown to correlate with pro-inflammatory cytokines (TNF-a; IL-6; C-reactive protein), either in experimental [22] or clinical research [13,26]. The presence of inflammation is a key component in the development and progression of VC [44] and the management of PD [2]. However, we did not find an association among inflammation, VC, and age in this population. Despite the fact that age and inflammation are well-known VC risk factors [4], VC is a complex and multifactorial process. In this sense, other variables, such as those involved in the calcium-phosphate metabolism, may overshadow the impact of age and hsCRP on VC in this population, and this phenomenon should be explored further.

Current guidelines state that inflammation and CVD risk stratification must be addressed to achieve successful CKD management [9,45]. Since we report a negative association between serum tGRP and VC, mineral metabolism, and inflammation markers, it is likely that tGRP may be a circulating marker reflecting VC burden. Thus, tGRP could be a promising tool for early and advanced VC diagnosis and monitoring in CKD management.

The strength of this study was the sample size of the study population. It represents a considerable dimension of patients in the context of peritoneal dialysis (*n* = 101), contributing to the clinical field by reporting VC diagnosis and other CVD risk parameters in a PD population.

This is the first clinical study addressing tGRP in the context of ESRD using a validated ELISA kit for determination of tGRP levels. Further, this is the first report demonstrating tGRP in peritoneal fluid. Although the relevance and molecular mechanism behind the presence of tGRP in peritoneal fluid is currently unknown, its positive correlation with tGRP serum levels suggests that it might be a direct consequence of solute transfer during dwell fluid exchange in PD sessions. Other proteins, like albumin, that have higher molecular weights than GRP, have been extensively reported to pass through blood capillaries into the dialysate [46]. The impact and implications of protein loss from circulation into the peritoneal fluid are currently under debate, and whether the presence of tGRP in 24 h dialysate represents a considerable loss of this protein was not investigated. A limitation of this study is that serum tGRP was measured at a single time point, without a time-course quantification during time in PD.

## 5. Conclusions

PD patients display decreased tGRP serum levels associated with VC and correlated with serum mineral and inflammation markers. Increased CVD risk (VCS ≥ 3) was found in 35.6% of PD patients with the lowest tGRP serum levels, while Ca, P, and VCS were found to be independent predictors for tGRP serum variability. Considering that mineral imbalance, VC, and inflammation are the main factors to consider during dialysis management, GRP might represent a potential CKD management tool, particularly as a VC marker with possible application in CVD risk assessment for CKD populations.

## Figures and Tables

**Table 1:** Characterization of the study population (*n* = 101) and Pearson’s correlation with tGRP serum levels.

Peritoneal Dialysis Patients			
Variables	Total (*n* = 101)	Pearson’s Correlation with Serum tGRP	
		r	*p* Value
Demographic			
Age, years	56.11 ± 1618	−0.153	0.116
Sex, m/f, *n* (male%)	62/39 (61.4)	NA	-
Biochemical (Serum Analysis)			
BMI, kg/m^2^	25.03 ± 0.53	−0.065	0.514
Total cholesterol, mg/dL	210 ± 26.21	0.016	0.831
HDL cholesterol, mg/dL	59 ± 1.87	0.024	0.712
LDL cholesterol, mg/dL	135 ± 30.10	0.091	0.614
Creatinine, mg/dL	12.17 ± 1.43	−0.151	0.081
P, mg/dL	5.11 ± 1.65	−0.482	0.001
Ca, mg/dL	7.88 ± 1.07	−0.503	<0.001
CaxP, mg^2^/dL^2^	36.67 ± 3.08	−0.575	<0.001
Mg, mg/dL	2.13 ± 0.39	0.242	0.015
Albumin, g/dL	3.98 ± 0.63	−0.052	0.605
Total Protein, g/dL	7.05 ± 0.10	0.204	0.061
tGRP, pg/mL (mean)	442.3 ± 34.02	NA	-
hsCRP, mg/L	8.82 ± 12.36	−0.313	<0.001
Dialysis and PET			
Time on PD, months	27.7 ± 2.97	0.135	0.242
Icodextrin Use, *n* (%)	54 (53.5)	NA	-
CADP (vs. APD), *n*(%)	33 (32.7)	NA	-
RKF, mL/min/1.73 m^2^	6.42 ± 3.54	−0.131	0.190
Creatinine Clear, L/1.73 m^2^/week)	97 ± 33.34	0.012	0.902
24 h Dialysate tGRP pg/mL	108 ± 10.21	0.397	<0.001
Vascular Calcifications	1.91 ± 2.18	−0.409	<0.001
VCS = 0, *n* (%)	41 (40.6)	NA	-
1 ≤ VCS ≤ 2, *n* (%)	24 (23.8)	NA	-
VCS ≥ 3, *n* (%)	36 (35.6)	NA	-

Data are presented as mean ± SD or number (frequency), as appropriate. *p* Value for Pearson’s correlations found between tGRP serum levels and demographic and biochemical assessments are given. Abbreviations: m/f, males/females; BMI, body mass index; P, phosphate; Ca, calcium; CaxP, calcium and phosphate product; Mg, magnesium; tGRP, total Gla-rich protein; hsCRP, high-sensitivity C-reactive protein; CADP, continuous ambulatory peritoneal dialysis; APD, automated peritoneal dialysis; RFK, residual kidney function; NA, not applied.

**Table 2:** Determinants of tGRP in serum in PD population (*n* = 101) based on linear regression.

Variables	tGRP Serum, pg/mL	
	B-Coefficient	*p* Value
P, mg/dL	−0.224	0.016
Ca, mg/dL	−0.422	<0.001
Mg, mg/dL	0.131	0.159
hsCRP, mg/L	−0.189	0.113
VCS	−0.229	0.011
	Model	
	r^2^ = 0.327	

The following exposures were entered: Ca, P, Mg, hsCRP, and VCS. Abbreviations: P, phosphate; Ca, calcium; Mg, magnesium; hsCRP, high-sensitivity C-reactive protein; VCS, vascular calcification score; tGRP, total Gla-rich protein.

**Table 3:** Associations found between 24 h dialysate and serum mineral and inflammatory markers.

Variables	Pearson’s Correlation with tGRP 24 h Dialysate, pg/mL	
	r	*p* Value
P, mg/dL	−0.235	<0.05
Ca, mg/dL	−0.252	<0.05
CaxP, mg^2^/dL^2^	−0.324	<0.01
Mg, mg/dL	0.172	0.071
hsCRP, mg/L	0.017	0.831

Pearson’s correlation coefficient (r), two tailed. Abbreviations: P, phosphate; Ca, calcium; CaxP, calcium and phosphate product; Mg, magnesium. hsCRP, high-sensitivity C-reactive protein; tGRP, total Gla-rich protein.

**Table 4:** Correlations between vascular calcification score (VCS) and clinical parameters of peritoneal dialysis patients.

Variables	Pearson’s Correlation with VCS	
	r	*p* Value
Demographic		
Age, years	0.083	0.393
BMI, kg/m^2^	0.131	0.183
Biochemical (Serum Analisys)		
Total cholesterol, mg/dL	0.245	0.159
HDL cholesterol, mg/dL	−0.151	0.343
LDL cholesterol, mg/dL	0.231	0.215
P, mg/dL	0.241	0.020
Ca, mg/dL	0.210	0.023
CaxP, mg^2^/dL^2^	0.301	0.001
Mg, mg/dL	0.189	0.145
Total Protein	−0.123	0.216
Serum tGRP, pg/mL (mean)	−0.409	<0.001
hsCRP, mg/L	0.140	0.160
Dialysis and PET		
Time on PD, months	−0.028	0.806
RKF, ml/min/1.73 m^2^	0.112	0.261
Creatinine Clear, L/1.73 m^2^/week)	−0.030	0.763
24 h Dialysate tGRP pg/mL (mean)	−0.212	0.036

Pearson’s correlation coefficient (r), two-tailed. Abbreviations: hsCRP, high-sensitivity C-reactive protein; BMI, body mass index; HDL, high density lipoprotein; LDL, low density lipoprotein; P, phosphate; Ca, calcium; CaxP, calcium and phosphate product; Mg, magnesium; tGRP, total Gla-rich protein; VCS, vascular calcification score.

**Table 5:** Biochemical variations of P, Ca, CaxP, and tGRP levels among vascular calcification score (VCS) groups.

Variables	Peritoneal Dialysis Patients			
	VCS, Adragão Method			*p* Value
	VCS = 0	1 ≤ VCS ≤ 2	VCS ≥ 3	
P, mg/dL	4.76 ± 1.51	4.78 ± 1.38	5.39 ± 1.56	0.014
Ca, mg/dL	7.50 ± 1.14	7.93 ± 0.9	8.31 ± 0.94	0.032
CaxP, md^2^/dL^2^	29.4 ± 18.4	36.5 ± 19.4	40.1 ± 15.1	0.012
Serum tGRP, pg/mL	785.1 ± 365.2	702.7 ± 301.1	419.4 ± 198.5	<0.001
24 h Dialysate tGRP, pg/mL	137.9 ± 114.0	103.8 ± 83.3	75.8 ± 90.4	0.211

Data are presented as mean ± SD. Differences between biochemical variables are given by *p* Values obtained using one-way ANOVA tests. Abbreviations: P, phosphate; Ca, calcium; CaxP, calcium and phosphate product; Mg, magnesium; tGRP, total Gla-rich protein; VCS, vascular calcification score.

**Table 6:** Differences among VCS groups using Tukey’s post hoc comparisons.

Variables	Significant Differences Between VCS Groups (*p* < 0.05)
P, mg/dL	VCS = 0 vs. VCS ≥ 3
Ca, mg/dL	VCS = 0 vs. VCS ≥ 3
CaxP, md^2^/dL^2^	VCS = 0 vs. VCS ≥ 3
Serum tGRP, pg/mL	VCS = 0 vs. VCS ≥ 3 and 1 ≤ VCS ≤ 2 vs. VCS ≥ 3

Data presents which specific group differences are significant for each variable, complementing the overall ANOVA results. Tukey’s post hoc test was performed to assess differences in serum measurements across clinical VCS score groups. Abbreviations: P, phosphate; Ca, calcium; CaxP, calcium and phosphate product; Mg, magnesium; tGRP, total Gla-rich protein; VCS, vascular calcification score.

## Data Availability

Data are available upon request due to privacy restrictions. The data presented in this study are not available, because they are in the process of analysis for result publication.
